# Global longitudinal strain at 3 months after therapy can predict late cardiotoxicity in breast cancer

**DOI:** 10.1002/cam4.6039

**Published:** 2023-05-15

**Authors:** Zhiyue Liu, Mei Liu, Xiaorong Zhong, Yupei Qin, Ting Liang, Ting Luo, Xi Yan, Zhuoqin Tang, Xi Wang, Shichu Liang, Qian Li, Xiaomiao Ruan, Wenfeng He, He Huang

**Affiliations:** ^1^ Department of Cardiology West China Hospital of Sichuan University Chengdu Sichuan China; ^2^ Department of Oncology West China Hospital of Sichuan University Chengdu Sichuan China; ^3^ Department of Ultrasound West China Hospital of Sichuan University Chengdu Sichuan China

**Keywords:** breast cancer, cardio‐oncology, echocardiography, heart failure, late‐onset cardiotoxicity

## Abstract

**Background:**

Cancer therapy‐related cardiovascular toxicity (CTR‐CVT) is a major contributor to poor prognosis in breast cancer (BC) patients undergoing chemotherapy. Left ventricular global longitudinal strain (LV GLS) has predictive value for CTR‐CVT, while few researchers take into account late‐onset CTR‐CVT. This study sought to provide a guide for the prediction of late‐onset CTR‐CVT in primary BC over the 2 years follow‐up via strain and contrast‐enhanced echocardiography.

**Methods:**

Anthracycline and anthracycline + targeted medication groups were created from 111 patients with stage I–III primary BC who were prospectively included. The left ventricular diastolic function, LV global long‐axis strain (GLS); left ventricular ejection fraction by contrast‐enhanced echocardiography (c‐LVEF), and electrocardiograms were collected at baseline, 3, 6, 12, and 24 months after the start of cancer treatment. The high‐sensitivity troponin‐T and NT‐pro BNP at baseline and 3 months after chemotherapy were measured.

**Results:**

(1) LV GLS decreased in BC patients over time. (2) After 12 months' follow‐up, the LV GLS in the anthracycline+ targeted group was lower than in the anthracycline group. After 24 months' follow‐up, the GLS and c‐LVEF in the anthracycline + targeted group declined while the E/e’ increased. (3) Decreased LVEF (56%) and arrhythmia (38%) are the common causes of CTR‐CVT. Lower LVEF was a major factor in late‐onset CTR‐CVT. (4) Combination of LV GLS and c‐LVEF at 3 months were used as predictors for CTR‐CVT and exhibited a higher AUC than either one alone (AUC = 0.929, 95% CI: 0.863–0.970). LV GLS at 3 months can predict the late‐onset CTR‐CVT (AUC = 0.745, *p* < 0.001), and the cut‐off is 20.32%.

**Conclusions:**

As time went on, the systolic and diastolic dysfunction of BC patients get worsened. The combination of LV GLS and c‐LVEF is better in the prediction of CTR‐CVT. Only the LV GLS at 3 months can predict the late‐onset CTR‐CVT.

## Graphical abstract

The poor prognosis of breast cancer (BC) patients is largely due to chemotherapy‐induced cardiotoxicity (CTIC). Few researchers take into account late‐onset CTR‐CVT. With a 2‐year follow‐up, the results of this study were used to build a more accurate and trustworthy strategy for managing CTIC risk variables. Regarding of the late‐onset CTR‐CVT, LV‐GLS at 3 months had a prediction value.

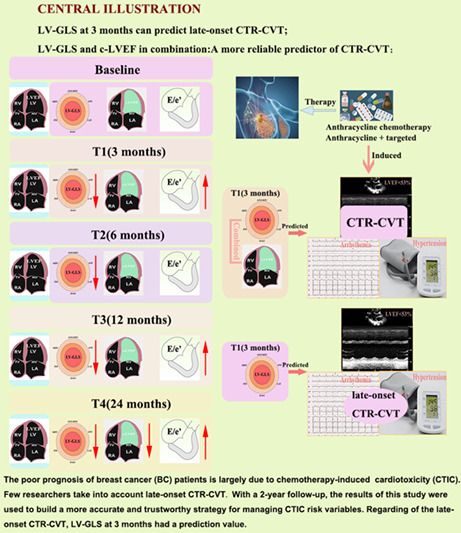

## 1 INTRODUCTION

Breast cancer (BC) accounts for 30% of newly diagnosed malignant tumors in women and causes 15% of cancer deaths in women.^1^ In BC treatment today, innovations like chemoradiotherapy, immunotherapy, and gene‐targeted therapy have been shown to extend overall survival. Nevertheless, Cancer therapy‐related cardiovascular toxicity definitions (CTR‐CVT)^2^, ^3^ emerge as a major contributor to poor prognosis in those undergoing chemotherapy. Furthermore, long‐term or “late” adverse effects of cancer treatments are increasingly important. Therefore, early detection of CTR‐CVT and late‐onset CTR‐CVT is necessary for early intervention.

Echocardiography is of helps to detect early impaired cardiorespiratory fitness. LVEF was suggested by several guidelines^4^ to detect CTR‐CVT; however, the endocardium is not well‐displayed for BC patients who underwent left breast surgery, dilator implantation and left thoracic radiotherapy. Contrast‐enhanced echocardiography as the use of ultrasound contrast agents based on conventional transthoracic echocardiography (TTE), can clearly depict the endocardial border and reduce interobserver variation in measuring LVEF.^5^ Suwatanaviroj et al.^6^ found that contrast‐enhanced echocardiography had a comparatively greater sensitivity for detecting changes in volume when the observed LVEF varied by no <3.6%.

Left ventricular global longitudinal strain (LV GLS) has predictive value for CTR‐CVT and HF and can detect early subclinical myocardial impairment related to cancer treatment,^4^, ^7^, ^8^ and can be used in the regular monitoring during and after treatment.^9^ According to a study of BC patients with a 6‐year follow‐up, 13.5% of participants had abnormal GLS, ranging from 11% to 15% among treatment groups. However, there are few studies examining the connection between early GLS alterations and late cardiotoxicity.

The objectives of this prospective cohort study were to evaluate the changes in the LV function of primary BC patients via GLS and contrast‐enhanced echocardiography, sought to provide a guide for the prediction of late‐onset CTR‐CVT (≥1 year) in primary BC over the 2 years follow‐up via strain and contrast‐enhanced echocardiography.

## 2 METHODS

### 2.1 Data source and study population

This study is a single‐center prospective cohort study from a registered study with the identifier ChiCTR1900022108 (http://www.chictr.org.cn). Consecutive patients with stage I‐III primary breast invasive ductal carcinoma receiving anthracycline chemotherapy or followed by sequential targeted therapy between May 2019 and December 2019 were enrolled. The patients were diagnosed with BC by Version 3. 2018, National Comprehensive Cancer Network (NCCN) Clinical Practice Guidelines.^10^ Inclusion criteria: (1) ≥18 years old; (2) LVEF ≥53% measured by biplane Simpson's rule by TTE before chemotherapy.^11^ Exclusion criteria include: ventricular bigeminy, atrial flutter, atrial fibrillation, and other severe arrhythmias; severe valvular disease, including moderate–severe aortic stenosis, moderate–severe mitral stenosis, and moderate–severe mitral regurgitation; myocardial infarction or symptomatic HF; history of cardiac surgery; use of cardiotoxic chemotherapy drugs in the past; and previous left thoracic radiotherapy. According to treatment procedures, the patients were assigned into two groups: anthracyclines group and anthracyclines + targeted therapy group. This study was approved by the Biomedical Ethics Committee of West China Hospital, Sichuan University (Approval Number: 20180517), and all patients provided informed consent.

### 2.2 Clinical evaluation

**FIGURE 1:**
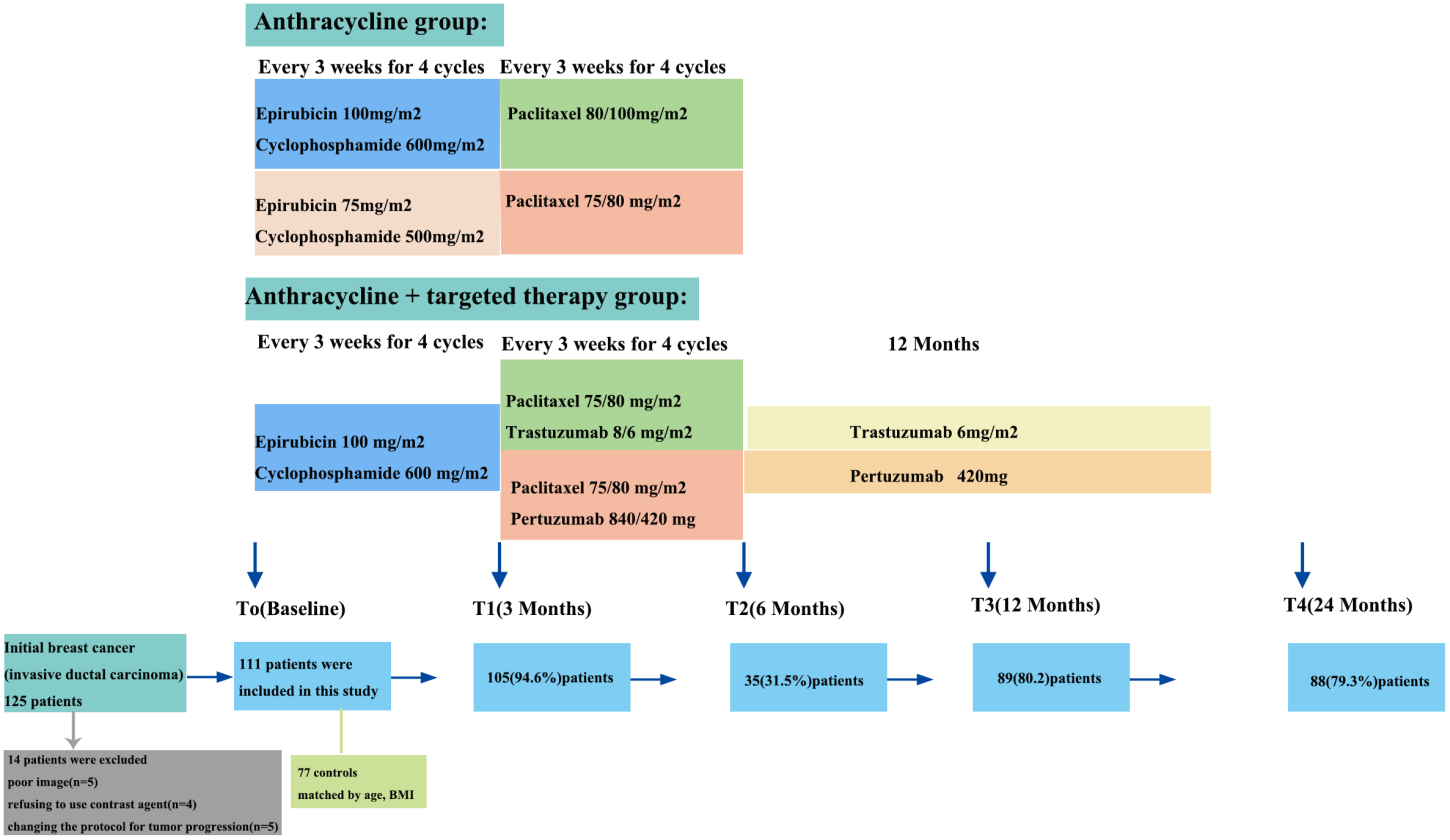
The treatment and follow‐up protocols of breast cancer patients.

The following information was collected: age, height, weight, systolic and diastolic blood pressure, history of chronic illness (hypertension and diabetes), tumor node metastasis (TNM) stage and endocrine therapy, and thoracic radiation. Holter was carried out if the electrocardiogram (ECG) revealed any new arrhythmias. At baseline (T0), follow‐up at 3 months (T1), 6 months (T2), 12 months (T3), and 24 months (T4) post‐chemotherapy, TTE and ECG were conducted. At T0 and T1, the myocardial injury biomarkers including NT pro‐BNP, troponin‐T, and creatine kinase isoenzyme (CK‐MB) were collected. Follow‐up terminated by January 31, 2022. Figure 1 displayed the follow‐up chart. The control group was comprised of 77 individuals who had completed normal ECG, TTE, and laboratory tests during the same period. The controls were used to compare the cardiac structure and function of patients before anthracycline‐based therapy, in order to exclude the possibility that surgery and the tumor itself had affected their cardiac function. In addition, we matched the prevalence of hypertension and diabetes between the case group and the control group since these diseases can also impact the LV GLS.

### 2.3 Echocardiography

The ultrasound system (Philip Epiq7C and Philip CVx) with a 1–5 MHz phased‐array transducer (M5S) was used. Standard echocardiography, including two‐dimensional (2D), M‐mode, and Doppler echocardiography, was performed according to the guidelines of the American Society of Echocardiography.^12^ LVEF was measured based on the modified biplane Simpson's rule. Mitral inflow velocity was measured at early (defined as E wave) and late (defined as A wave) diastole. The velocity of the mitral annulus at early diastolic (defined as e’) and late diastolic (defined as a’) was recorded by pulsed tissue Doppler imaging. The E/e’ ratio was used as an index of LV diastolic function. All images were captured by a senior operator.

**FIGURE 2:**
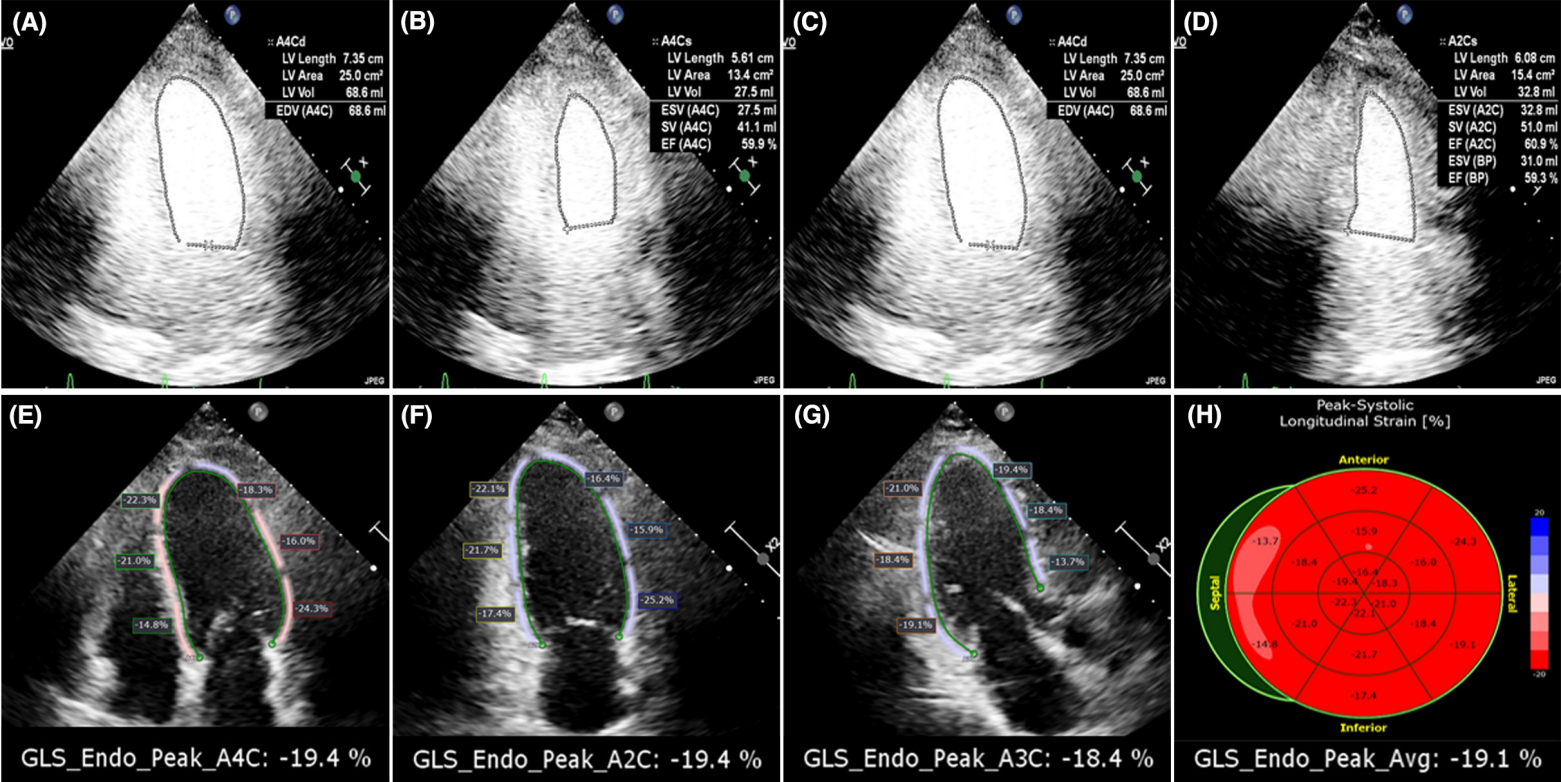
(A–D) The measurement of c‐LVEF by biplane Simpson's rule; (E–H) The measurement of LV GLS by Autostrain.

**FIGURE 3:**
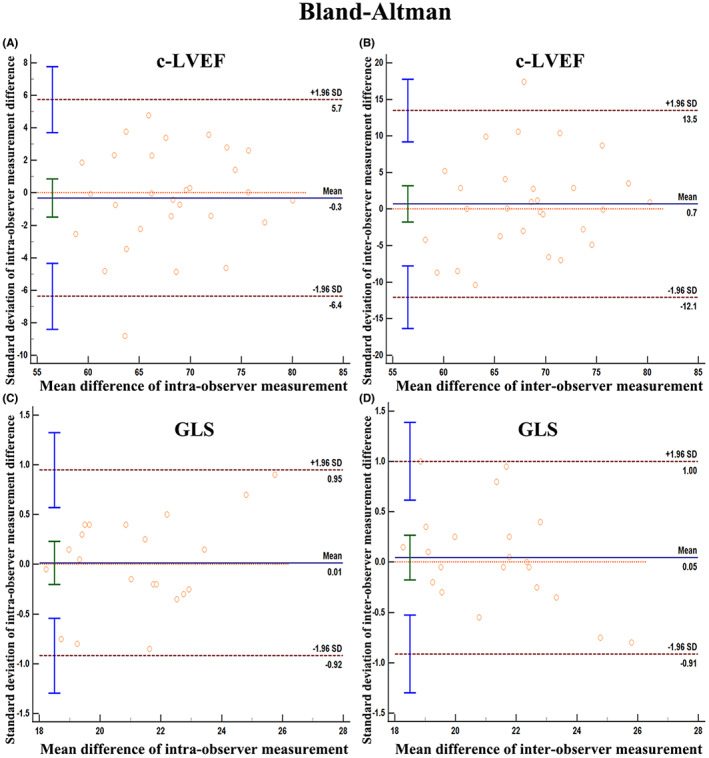
(A) The intra‐observer variability in c‐LVEF measurements; (B) The inter‐observer variability in c‐LVEF measurement. (C) The intra‐observer variability in GLS measurements; (D) The inter‐observer variability in GLS measurements.

Apical four‐, two‐, and three‐chamber images of at least five cardiac cycles were captured and used to calculate the LV GLS. The strain analytical software (AutoStrain, Philips) automatically detects, segments, and monitors LV myocardium after selecting the region of interest for examination, with strain curves being computed simultaneously. To provide a good portrayal of ventricular motion, every track was examined. Poorly tracked segments were manually corrected until a higher tracking quality was attained. When more than two portions of a video could not be tracked satisfactorily, the video was deemed to have “failed”.^13^ All cases were analyzed using the Auto Strain software (Philips Healthcare, Figure 2E–H). The analysts were blinded to the measurements performed and the clinical data. Bland–Altman analysis showed good intra‐ and inter‐observer agreement with a small nonsignificant bias for GLS (Figure 3C,D).

### 2.4 Contrast‐enhanced echocardiography

All patients received an intravenous injection of Sono vue (Bracco SpA). Apical four‐, three‐, and two‐chamber images of at least five cardiac cycles were digitally recorded and archived for analysis. LVEF (designated as c‐LVEF) was estimated from the contrast recordings in the Philips system using the biplane Simpson's rule (Figure 2A–D). Bland–Altman analysis showed good intra‐ and inter‐observer agreement with a small nonsignificant bias for c‐LVEF (Figure 3A,B).

### 2.5 Cancer therapy‐related cardiotoxicity

CTR‐CVT includes cancer treatment‐related cardiac dysfunction (CTRCD), which is defined by the European Society of Cardiology guidelines as having an LVEF<50% or a decrease of >10% post‐chemotherapy, or a new GLS decrease of 15% relative to baseline, immune checkpoint inhibitor‐induced myocarditis, cancer treatment‐related vascular toxicity, cancer treatment‐related arterial hypertension, and cancer treatment‐related arrhythmia.^14^ Early‐onset CTR‐CVT includes acute cardiotoxicity which can occur during or soon after initiation of therapy and occurs within the first year of completion of chemotherapy, whereas Late‐onset CTR‐CVT occurs beyond the first year and up to 2 years in our study.^15^

### 2.6 Statistical analysis

For normally distributed data, continuous variables were displayed as mean standard deviation (SD) or median (25th percentile and 75th percentile) for non‐normally distributed data. Frequencies were used to present categorical variables. For comparing two and three independent groups of normally distributed data, the *t*‐test, and one‐way analysis of variance (ANOVA) were used, respectively. The non‐normal distribution was tested using the Wilcoxon signed‐rank test. Binary variables were compared using Fisher's exact test and the Chi‐square test. To evaluate the risk factors of CTR‐CVT in more detail, multivariable logistic regression models were applied. The multivariate regression analysis model included contained univariate regression analysis of positive factors. After accounting for echocardiographic indices, a receiver operating characteristic (ROC) curve analysis was carried out. Bland–Altman plots were used to display the difference in intra‐observer and inter‐observer of c‐LVEF and GLS, along with the average bias and 95% limits of agreement. *p* < 0.05 were taken into consideration. IBM SPSS for Windows, version 19.0 (IBM Corp) was used for all analyses.

## 3 RESULTS

### 3.1 General data of patients

Fourteen patients were removed, as in Figure 1, including poor image quality (*n* = 5), reluctance to contrast (*n* = 4), and renewed treatment regimen due to distant metastasis (*n* = 5). Finally, this study enrolled a total of 111 patients and 77 controls. At 3 months (T1), 105 patients (94.6%) were on follow‐up, 35 patients (31.5%) at 6 months (T2), 89 patients (80.2%) at 12 months (T3), and 88 patients (79.3%) at 24 months (T4).

Age, body mass index (BMI), chronic diseases like hypertension, and diabetes, tumor node metastasis (TNM) stage, and other treatment indicators for BC did not significantly alter between baseline and follow‐up (Table 1).

**TABLE 1:** The general data and echocardiography index of primary breast cancer patients and controls.

	Controls (*n* = 77)	T0 (*n* = 111)	T1 (*n* = 105)	T2 (*n* = 35)	T3 (*n* = 89)	T4 (*n* = 88)	*p*‐Value
Age (years)	49.52 ± 11.98	48.39 ± 10.12	48.52 ± 10.24	45.37 ± 10.09	47.03 ± 10.09	47.40 ± 10.02	0.379
BMI (kg/m^2^)	23.24 ± 2.89	23.35 ± 3.41	23.24 ± 3.37	23.03 ± 2.93	23.31 ± 3.51	23.38 ± 3.30	0.996
Hypertension	8 (10.4%)	7 (6.3%)	7 (6.7%)	2 (5.7%)	6 (6.7%)	6 (6.8%)	0.910
Diabetes	2 (2.6%)	3 (2.7%)	3 (2.9%)	2 (5.7%)	2 (2.2%)	2 (2.3%)	0.915
TNM stages (I/II/III)	/	14/47/50	12/44/49	7/15/13	11/40/38	11/38/39	0.970
Targeted drugs	/	28 (25.2%)	28 (26.7%)	12 (34.3%)	22 (24.7%)	22 (25.0%)	0.783
Radiotherapy (2GY)	/	61 (55.0%)	59 (56.2%)	23 (65.7%)	52 (58.4%)	53 (60.2%)	0.808
Endocrine therapy	/	46 (41.4%)	46 (43.8%)	13 (37.1%)	41 (46.1%)	40 (45.5%)	0.887
RV (mm)	20.64 ± 2.05	20.91 ± 2.55	20.94 ± 2.72	20.97 ± 2.33	20.93 ± 2.41	20.81 ± 2.50	0.964
IVS (mm)	8.74 ± 1.33	8.71 ± 1.55	9.21 ± 1.84	9.31 ± 1.88	9.33 ± 1.78*	9.68 ± 1.88*	0.001
LV (mm)	44.90 ± 3.07	43.55 ± 4.06	43.46 ± 3.95	43.63 ± 4.34	43.12 ± 4.48	43.72 ± 3.91	0.081
LVPW (mm)	8.22 ± 0.96	8.09 ± 1.95	8.35 ± 2.09	8.20 ± 1.51	8.37 ± 1.46	8.53 ± 1.35	0.533
E/e’	7.43 ± 2.25	7.21 ± 1.92	8.12 ± 2.38*	8.00 ± 2.49	8.15 ± 2.51*	8.63 ± 2.79*	0.001
LVEF (%)	60.82 ± 5.77	59.33 ± 6.66	59.87 ± 6.47	61.35 ± 7.29	59.82 ± 6.57	59.28 ± 7.16	0.443
c‐LVEF (%)	/	60.68 ± 5.11	60.53 ± 5.48	60.09 ± 6.25	60.02 ± 5.99	58.74 ± 5.95*	0.046
LV GLS (%)	22.28 ± 2.06	23.11 ± 3.14	21.25 ± 3.21*	21.67 ± 2.79*	20.29 ± 2.84*#	19.31 ± 2.93*#	<0.001

Abbreviations: BMI, body mass index; TNM, tumor node metastasis.

* Compared with baseline, *p* < 0.05.

# Compared with 3 months’ follow‐up, *p* < 0.05.

### 3.2 Cardiac structure and function

At baseline, there was no discernible difference between controls and patients in terms of structure and function (Table 1). No difference in cardiac chamber's size at follow‐up either. LV GLS decreased at T1, T2, T3, and T4 compared to baseline (*p* < 0.05). When compared to LV GLS at 3 months (T1) follow‐up, LV GLS at 1 year (T3) and 2 years (T4) decreased. E/e’ increased at 3 months (T1), 1 year (T3), and 2 years (T4) compared to baseline (T0). Additionally, when compared to baseline (T0) and 3 months (T1), serum myocardial injury biomarkers, such as CK‐MB, increased at 6 months (T2) (*p* < 0.05) (Table 2). These findings implied that myocardial injury may occur after a 6‐month pharmaceutical regimen, and subclinical systolic dysfunction may occur at 3 months.

**TABLE 2:** The cardiac injury biomarkers in the follow‐up of primary breast cancer patients.

	T0 (*n* = 110)	T1 (*n* = 55)	T2 (*n* = 73)	*p‐*Value
Myoglobin (ng/mL)	24.87 ± 23.10	22.43 ± 9.09	25.52 ± 6.80	0.544
CK‐MB (ng/mL)	0.93 ± 0.37	0.86 ± 0.33	1.20 ± 0.77^a^, ^b^	0.045
NT‐pro BNP (ng/mL)	65.51 ± 59.15	74.09 ± 77.90	44.38 ± 47.98	0.439
troponins‐T (ng/mL)	6.31 ± 2.63	5.47 ± 2.79	5.30 ± 6.63	0.222

^a^ Compared with baseline, *p* < 0.05.

^b^ Compared with 3 months' follow‐up, *p* < 0.05.

After a year of follow‐up, GLS in the anthracycline + targeted group decreased in comparison to the anthracycline group (*p* < 0.05), but the c‐LVEF showed no difference (Table 3). The GLS and c‐LVEF both decreased after 2 years of follow‐up, while the E/e’ rose (*p* < 0.05) in the anthracycline + targeted group compared to the anthracycline group.

**TABLE 3:** The left ventricular function in the anthracycline group and anthracycline + targeted therapy group at follow‐up.

	Anthracycline	Anthracycline + targeted therapy	*p*‐Value
Baseline (T0)	*N* = 84	*N* = 27	
GLS	23.03 ± 3.20	23.33 ± 3.00	0.639
c‐LVEF	60.56 ± 5.37	61.03 ± 4.30	0.704
E/e’	7.40 ± 1.88	6.66 ± 1.77	0.157
3 months (T1)	*N* = 78	*N* = 27	
GLS	21.49 ± 3.34	20.61 ± 2.76	0.184
c‐LVEF	60.38 ± 5.60	60.95 ± 5.23	0.645
E/e’	8.36 ± 2.52	7.50 ± 1.86	0.105
6 months (T2)	*N* = 23	*N* = 12	
GLS	21.36 ± 2.62	22.27 ± 3.13	0.397
c‐LVEF	61.14 ± 6.52	58.09 ± 5.40	0.125
E/e’	8.38 ± 2.64	7.29 ± 2.10	0.201
1 year (T3)	*N* = 67	*N* = 22	
GLS	21.73 ± 2.82	19.96 ± 2.51	0.016
c‐LVEF	59.97 ± 5.94	60.15 ± 6.29	0.896
E/e’	8.22 ± 2.53	7.92 ± 2.49	0.606
2 years (T4)	*N* = 66	*N* = 22	
GLS	19.95 ± 2.78	17.39 ± 2.52	0.001
c‐LVEF	59.80 ± 5.11	55.55 ± 7.19	0.003
E/e’	8.29 ± 2.55	9.66 ± 3.26	0.020

Abbreviations: GLS, global longitudinal strain; LVEF, left ventricular ejection fraction.

### 3.3 Cancer therapy‐related cardiotoxicity in BC patients

**FIGURE 4:**
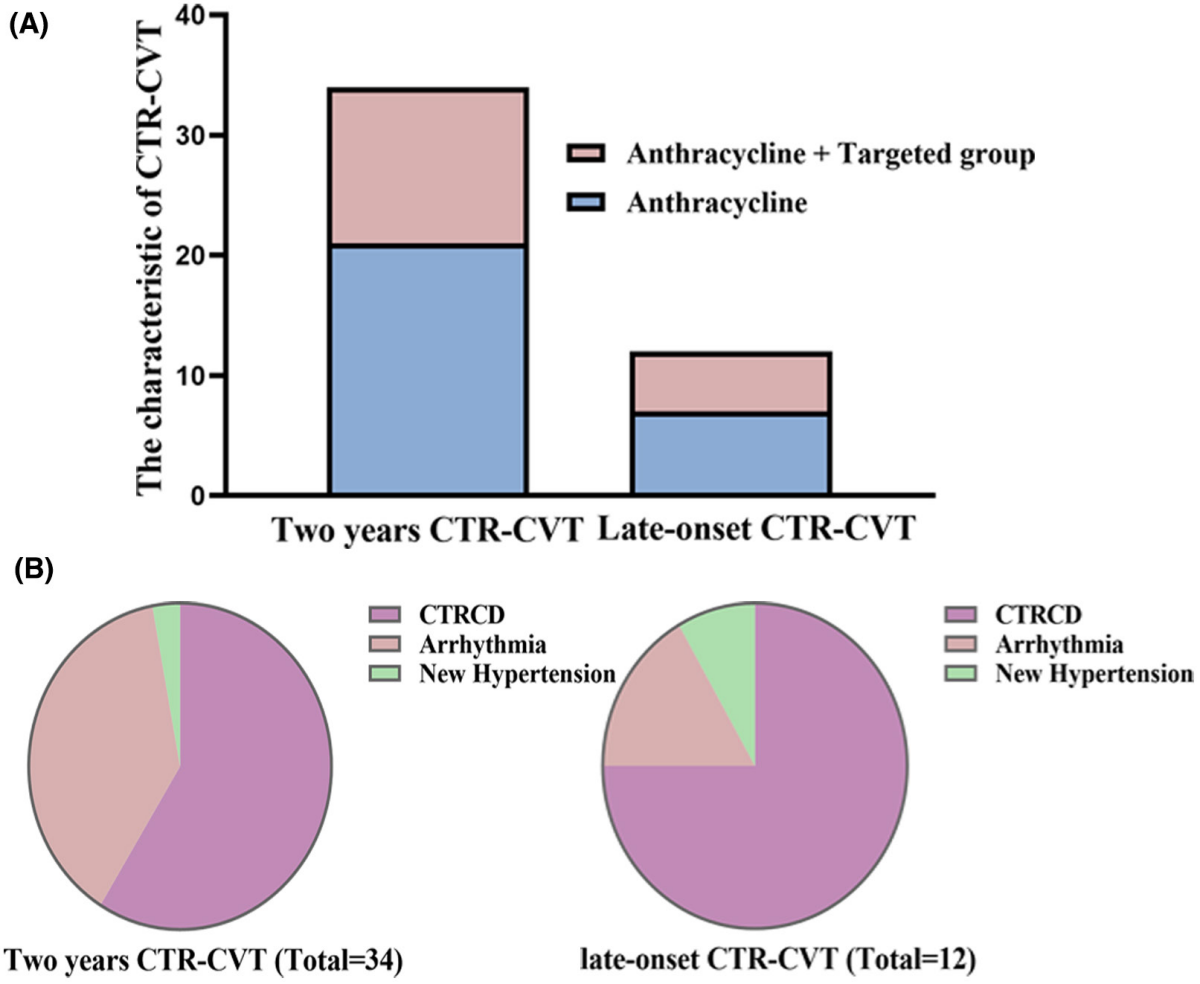
(A) The characteristics of the CTR‐CVT in anthracycline and anthracycline + targeted group (The Y‐axis represents the number of CTR‐CVT cases and the X‐axis represents the time of occurrence of CTR‐CVT.); (B) Constitution diagram of CTR‐CVT.

The occurrence of CTR‐CVT was regarded as the endpoint event. A total of 34 patients had CTR‐CVT during the follow‐up, among them, 20 with CTRCD (5 patients with LVEF<50%, 7 patients with decreased LVEF by >10%, 8 patients with declined GLS by >15%), 13 arrhythmias, and 1 with newly developed hypertension. Twelve patients had late‐onset CTR‐CVT, including nine with decreased LVEF, two with arrhythmia, and one with newly developed hypertension. Figure 4 displayed the CTR‐CVT's proportion.

### 3.4 Factors of prediction for CTR‐CVT

**FIGURE 5:**
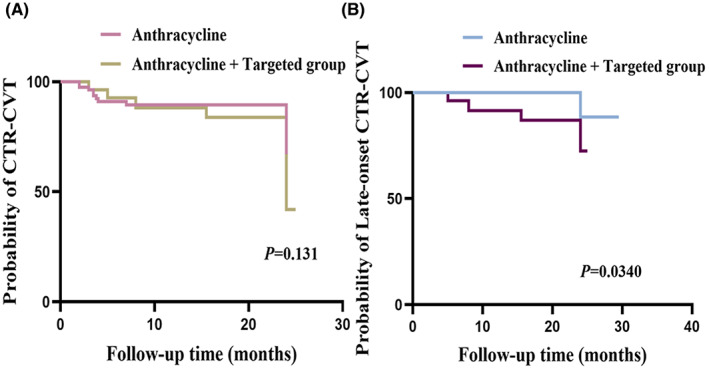
(A) The probability of CTR‐CVT during the 2 years' follow‐up between the anthracycline and anthracycline + targeted therapy; (B) The probability of late‐onset CTR‐CVT between the anthracycline and anthracycline + targeted therapy.

Plots illustrating the cumulative incidence of CTR‐CVT for the three groups—without CTR‐CVT, with CTR‐CVT, and with late‐onset CTR‐CVT—were made (Table 4). No difference in Age, systolic, and diastolic blood pressure, the proportion of patients receiving radiation, left thoracic radiotherapy, cumulative dose of anthracyclines (400/300 mg/m), and endocrine therapy, TNM stages, hypertension, or diabetes among all three groups, but targeted therapy was significantly different. While there was no difference in the 2‐year incidence of CTR‐CVT between the anthracycline and anthracycline + targeted groups, Kaplan–Meier analysis (Figure 5) revealed that the late‐onset CTR‐CVT was more common in the anthracycline + targeted group than in the anthracycline group (*p* = 0.034). Furthermore, we found that left thoracic radiotherapy can affect the early‐onset CTR‐CVT (*p* = 0.009). Out of a total of 22 patients with early‐onset CTR‐CVT, 12 patients had received left chest radiotherapy.

**TABLE 4:** The risk factors for CTR‐CVT in BC patients.

	No CTR‐CVT (*n* = 77)	CTR‐CVT (*n* = 34)	late‐onset CTR‐CVT (*n* = 12)	*p‐*Value
Age (year)	48.74 ± 9.85	47.59 ± 10.95	46.17 ± 10.78	0.669
BSA (kg/m^2^)	1.60 ± 0.11	1.60 ± 0.12	1.64 ± 0.14	0.448
SBP (mmHg)	117.64 ± 12.51	117.00 ± 11.38	118.5 ± 12.97	0.930
DBP (mmHg)	76.01 ± 10.18	74.85 ± 10.40	74.75 ± 8.00	0.818
Targeted drug	15	13^a^	6^a^	0.024
TNM (I/II/III)	9/34/34	5/16/13	2/4/6	0.906
Hypertension	5	2	1	0.957
Diabetes	1	1	0	0.952
Cumulative dose of anthracyclines (400/300 mg/m)	62/15	29/5	10/2	0.878
Endocrine therapy	30	16	8^a^	0.076
Radiotherapy	42	19	5	0.675
History of left thoracic radiotherapy	20	15	3	0.164

^a^ Compared with No CTR‐CVT, *p* < 0.05.

Univariate logistic regression analysis was used to examine potential risk variables for CTR‐CVT. The multivariate regression analysis model took the data with discrepancies into account (Table 5). The model demonstrated that in BC patients, 3‐month GLS, and c‐LVEF were independent risk factors for CTR‐CVT. Three separate risk factors for late‐onset CTR‐CVT included the 3‐month GLS, E/e’, and the proportion of the targeted medication.

**TABLE 5:** The risk factors for CTR‐CVT in patients with primary breast cancer.

	Univariate logistic regression analyses			Multivariate logistic regression analyses		
	OR	95% confidence interval	*p‐*Value	OR	95% confidence interval	*p‐*Value
CTR‐CVT						
LV GLS (baseline)	1.085	0.812–1.451	0.580			
c‐LVEF (baseline)	0.898	0.828–0.975	0.010	0.899	0.806–1.003	0.057
E/e’ (baseline)	1.071	0.869–1.320	0.517			
c‐LVEF (3 months)	0.865	0.798–0.939	0.001	0.765	0.659–0.889	<0.001
LV GLS (3 months)	0.554	0.441–0.696	0.001	0.470	0.340–0.649	<0.001
E /e’ (3 months)	0.944	0.803–1.108	0.480			
Targeted drug	0.433	0.177–1.060	0.067			
Late‐onset CTR‐CVT						
LV GLS (baseline)	1.002	0.829–1.212	0.981			
c‐LVEF (baseline)	0.961	0.861–1.071	0.470			
E/e’ (baseline)	0.847	0.599–1.199	0.350			
c‐LVEF (3 months)	0.953	0.867–1.047	0.319			
LV GLS (3 months)	0.614	0.474–0.795	0.001	0.578	0.427–0.783	<0.001
E/e’ (3 months)	1.223	1.003–1.490	0.046	1.368	1.012–1.849	0.042
Targeted drug	0.137	0.037–0.502	0.003	0.181	0.036–0.909	0.038

Abbreviations: GLS, global longitudinal strain; LVEF, left ventricular ejection fraction.

**FIGURE 6:**
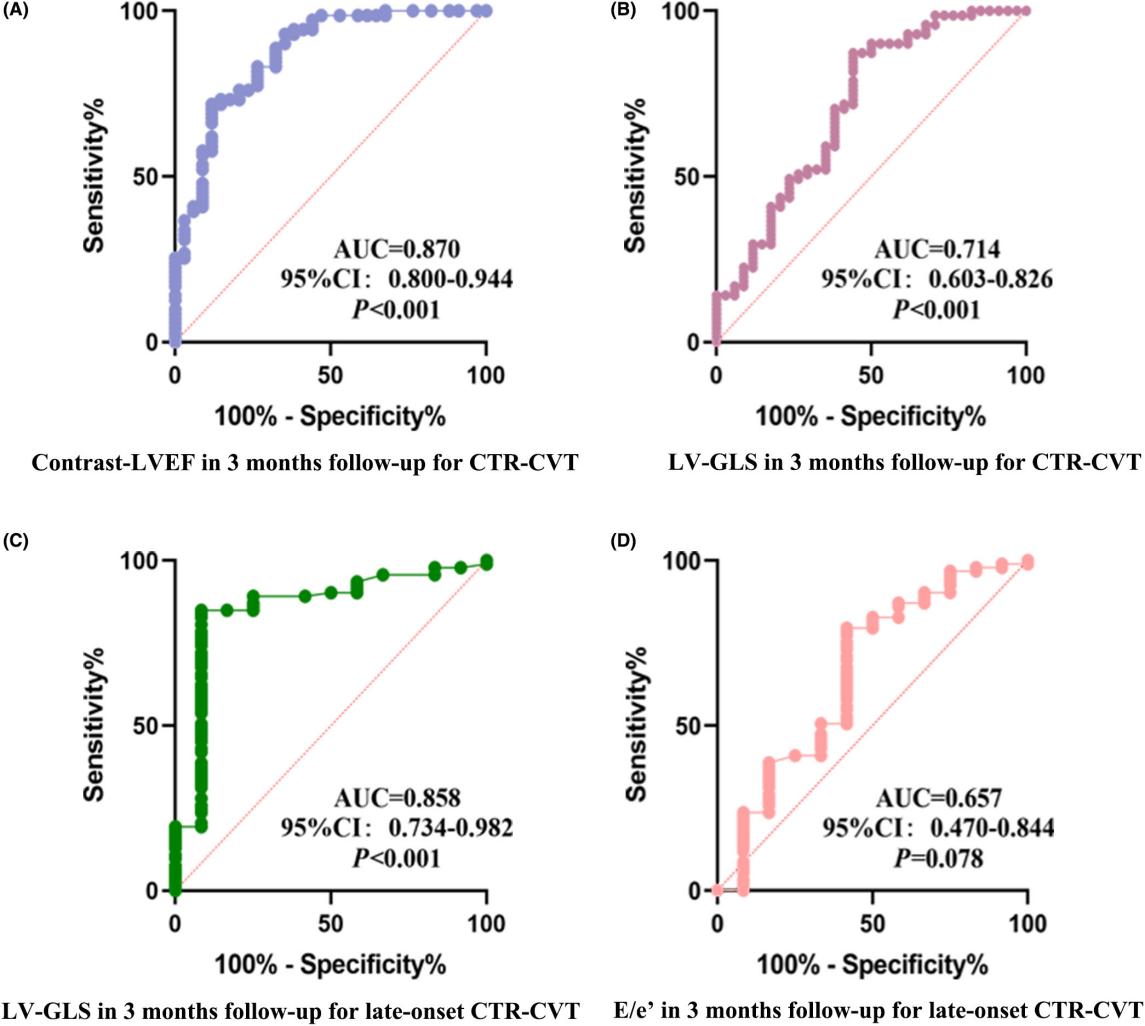
(A) The ROC curve of c‐LVEF in 3 months follow‐up predicts CTR‐CVT. (B) The ROC curve of LV‐GLS in 3 months follow‐up predicts CTR‐CVT. (C) The ROC curve of LV‐GLS in 3 months follow‐ups predicts late‐onset CTR‐CVT. (D) The ROC curve of E/e’ in 3 months follow‐ups predicts late‐onset CTR‐CVT.

**FIGURE 7:**
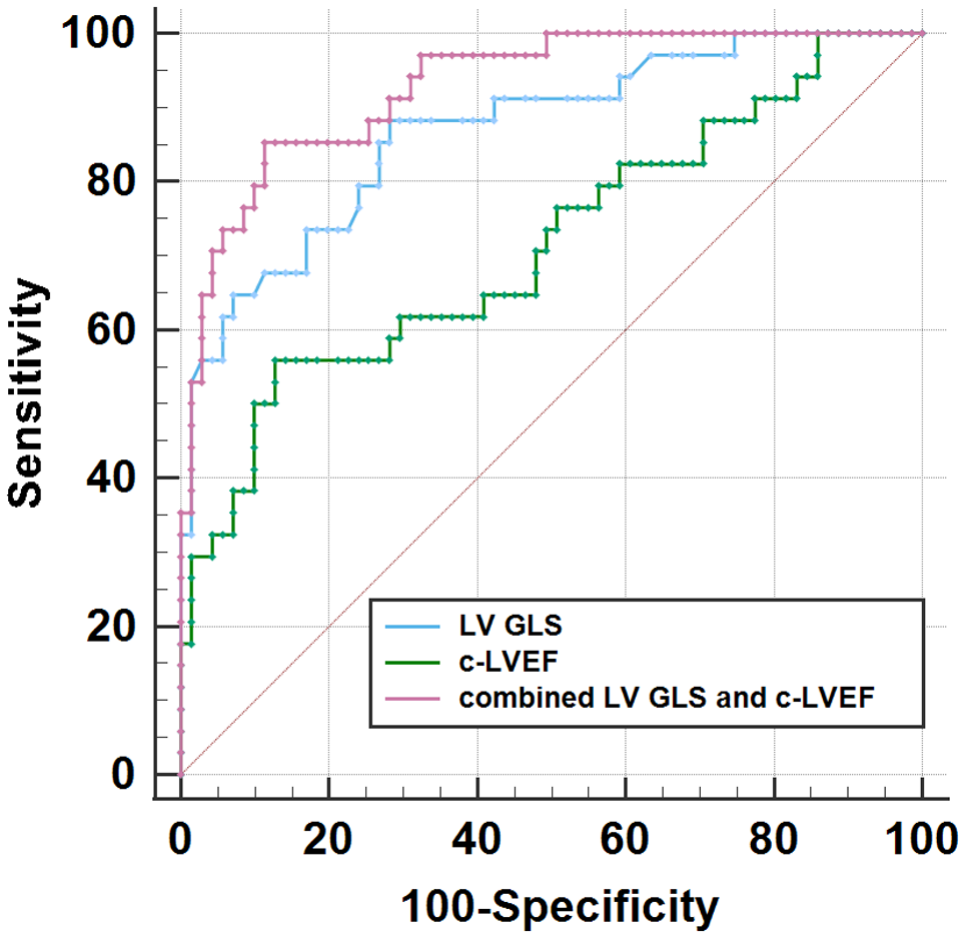
The ROC curve of combined GLS and c‐LVEF at 3 months' follow‐up predicts CTR‐CVT.

GLS (AUC = 0.870, *p* < 0.001) and c‐LVEF (AUC = 0.714, *p* < 0.001) at 3 months predicted CTR‐CVT according to ROC curve analysis (Figure 6). While the AUC of 3 months E/e’ for late‐onset CTR‐CVT showed no statistically significant difference (*p* > 0.05). GLS at three‐month predicted late‐onset CTR‐CVT (AUC = 0.745, *p* < 0.001). In addition, the cutoffs for the GLS at 3 months for predicting CTR‐CVT and late‐onset CTR‐CVT were 20.96% and 20.32%, respectively. The c‐LVEF cutoff for the treatment's anticipated CTR‐CVT at 3 months was 54%. When combination of LV GLS and c‐LVEF were used as predictors, the ROC curve in Figure. 7 exhibited a considerably higher AUC than either one alone (AUC = 0.929, 95% CI: 0.863–0.970).

## 4 DISCUSSION

**FIGURE 8:**
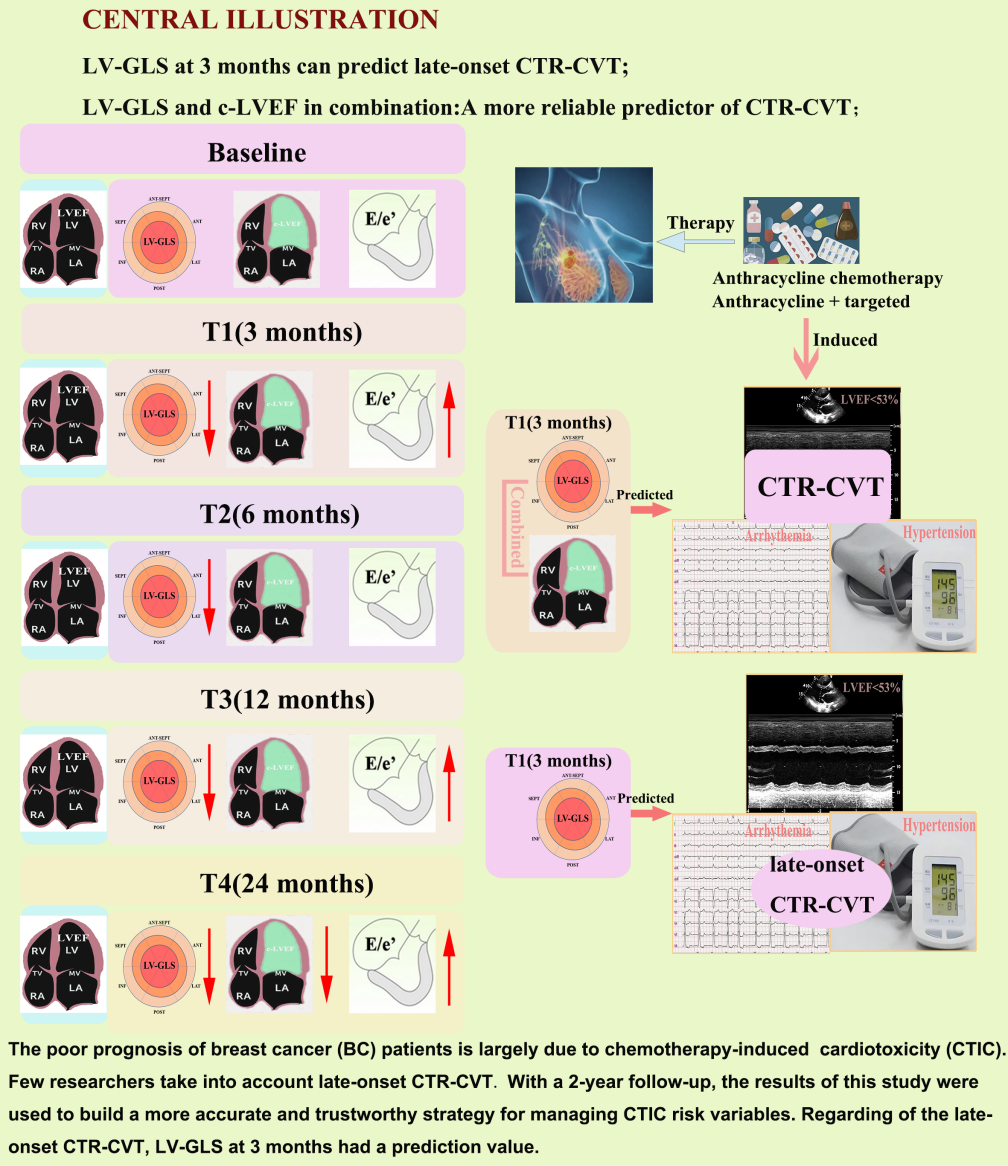
Central illustration LV‐GLS at 3 months can predict late‐onset CTR‐CVT; LV‐GLS and c‐LVEF in combination: a more reliable predictor of CTR‐CVT.

Our major findings can be summarized as follows (Figure 8): (i) In BC patients, LV GLS may decline over time; the E/e’ increased at the 3‐month follow‐up and stayed steady throughout 2‐year follow‐up. (ii) After a year of follow‐up, the anthracycline + targeted group's LV GLS was lower than that of the anthracycline group. The GLS and c‐LVEF dropped whereas the E/e’ increased in the anthracycline + targeted group after a two‐year follow‐up. (iii) A total of 34 patients experienced CTR‐CVT, with 12 of those experiencing late‐onset CTR‐CVT. (iv) Combination of LV GLS and c‐LVEF at 3 months were used as predictors for CTR‐CVT and exhibited a higher AUC than either one alone (AUC = 0.929, 95% CI: 0.863–0.970). LV GLS at 3 months can predict the late‐onset CTR‐CVT (AUC = 0.745, *p* < 0.001), and the cut‐off is 20.32%.

The 2‐year follow‐up of this study showed that the incidence of CTR‐CVT including early‐onset and late‐onset was 30.6% in primary BC patients, which was commensurate with the incidence of cardiotoxicity events (3%–35%) described in earlier studies.^2^, ^16^, ^17^ The difference in the definition of the incidence of cardiotoxicity events across studies is also not negligible. As stated in the literature, early‐stage CTR‐CVT was characterized by a high rate of cardiac arrhythmias, but late‐onset CTR‐CVT was characterized by a reduced LVEF.^18^ Previous studies also showed among patients who developed late severe cardiovascular events, 66 (48.5%) had HF, 40 (29.4%) cardiomyopathy, 19 (14.0%) myocardial injury, and 11 (8.1%) cardiovascular death.^19^ So it is still clinically necessary to identify individuals who are at a high risk of developing CTR‐CVT. If CTR‐CVT is discovered early, cancer treatments may be altered to reduce additional myocardial injury. In this context, imaging methods and biomarkers are required to identify at‐risk patients prior to a CTR‐CVT. Contrast‐enhanced echocardiography and GLS was adopted to assess left ventricular function in this study. For LVEF measures, a stronger association between contrast‐enhanced echocardiography and CMR was discovered.^20^ In the BC patients in our study, c‐LVEF decreased after a two‐year follow‐up, and c‐LVEF at 3 months (T1) can predict development of CTR‐CVT. Over the past 10 years, GLS has become a reliable indicator of subclinical LV dysfunction and is more sensitive than LVEF at detecting early changes in LV function.^21^ The published study with a median follow‐up of 24.7 months has shown that left ventricular ejection fraction, strain, and strain remain decreased at follow‐up, which was consistent with our study.^22^ This might be due to the persistent cardiac impairment of the chemotherapy drugs. In our study, GLS allowed an early detection of subclinical LV dysfunction and had predictive value for CTR‐CVT and late‐onset CTR‐CVT. Few studies also investigated the clinical value of 3‐month GLS, the cutoff values of LV GLS for predicting CTR‐CVT for 3 months range from −18.0% to −16.6%.^23^, ^24^ It was different with our 20.96% in CTR‐CVT and 20.32% in late‐onset CTR‐CVT, which might be related to different machines or our patients taking dexrazoxane. Overall, we found that a combination of GLS and c‐LVEF predicted CTR‐CVT best. To our knowledge, this is the first study to show the combination of GLS and c‐LVEF in the prediction of BC patients' outcomes.

In the 3‐month follow‐up, the E/e’ increased concurrently, implying diastolic dysfunction. Additionally, 3‐months E/e’ predicted BC patients' late‐onset CTR‐CVT. Unfortunately, because of the limited sample size, we did not get a positive result by ROC. Anthracyclines can affect diastolic function.^16^, ^25^ Abnormal diastolic function was associated with an increased risk of cancer‐treated reduced cardiac dysfunction.^16^ This was in line with our study. However further investigation is required in a large‐sample study to confirm the prediction value of E/e’.

We assessed serum cardiac markers including myoglobin, troponin T, and CK‐MB at baseline, 3 months, and 6 months after anthracycline‐based therapy. Previous research has shown that CK‐MB levels also increase during chemotherapy but with a different time course. In the anthracycline + targeted group, CK‐MB levels peaked at 3 months and then stabilized at 6 months and 1 year, while in HER2−patients, CK‐MB levels behaved similarly to the other biomarkers, with a slight increase at 3 months, a peak at 6 months, and a stabilization at 1 year after treatment.^26^ Therefore, we analyzed both troponin T and CK‐MB levels to assess the extent of myocardial damage. Based on previous studies, NT‐proBNP has been shown to have predictive value in the early diagnosis of cardiotoxicity. However, there are certain biological variations and factors such as age, body mass index, and renal function that need to be considered when interpreting the biomarker results. It is also important to note that earlier studies were limited by small sample sizes and the validity of cardiac assessments. Ponde et al.^27^ conducted an analysis using data from 280 patients with early breast cancer treated in the Neo‐ALTTO trial to assess the predictive power of NT‐proBNP. They found that elevations in biomarker levels were rare and failed to establish an association between these markers and cardiac events. Another study showed that when patients were dichotomized based on meeting CMR CTRCD criteria, there were no increased diagnostic values via hsTnI and BNP.^28^ It is important to consider that NT‐proBNP is an indicator of cardiac strain or volume overload and may not detect early cell injury. In our study, we found no difference in the size of the cardiac chamber and the cardiac systolic function among T0–T2, which may explain the lack of change in NT‐pro BNP until 6 months.

The cumulative dose of doxorubicin was not significantly linked with cardiotoxic events in our investigation, which is consistent with Slamon et al.'s findings [12], despite earlier studies reporting that anthracycline‐induced myocardial injury was dose‐dependent.^29^ This may be because the risk of developing cardiotoxicity is still low at doses below the higher recommended range of 900–1000 mg/m^2^.^30^ Anthracycline + targeted therapy can lead to changes in cardiac function in primary BC patients.^31^ In this study, anthracycline + targeted treatment was associated with higher cardiotoxicity events than anthracycline chemotherapy alone. 20%–45% of patients and 3% of patients, respectively, have heart failure or cardiotoxicity as a side effect of their targeted therapy.^32^ There is a distinct element of long‐term, irreversible damage to the myocardium,^33^ and cardiotoxicity is closely related to treatment stoppage, underscoring the seriousness of the problem, even though some of this damage is thought to be reversible following trastuzumab cessation.^22^ Previous studies indicated that cardiac radiation doses were associated with subclinical LV dysfunction evaluated by LV GLS. Some studies also showed through 40 patients treated for left breast cancer, that for a mean heart dose of 1.3–2.5Gy, there was no correlation between GLS and mean heart dose.^34^ In our study, there no difference in radiation history among the no CTR‐CVT, CTR‐CVT, and late‐onset CTR‐CVT group. However, there was a significant difference in the history of left chest radiation exposure between the early‐onset CTR‐CVT group and the non‐early‐onset CTR‐CVT group. Previous studies have also indicated that in patients with left‐sided breast cancer, GLS was reduced from −18.3% ± 3.1% to −17.2% ± 3.3% after undergoing radiation therapy for 6 months.^35^ Thus, compared to late‐onset CTR‐CVT, left chest radiation may more easily induce CTR‐CVT within the first year after therapy.

There are several limitations of our study. The early stages of the COVID‐19 outbreak in 2020 (January to June) had an impact on follow‐up rates. As a result, only a small number of BC patients returned to the hospital for follow‐up at T2, which is a major limitation of this study. A larger sample size would have allowed us to draw more conclusive results, which could present a statistical limit in multivariate analysis and ROC curve.

## 5 CONCLUSIONS

As time went on, the systolic and diastolic dysfunction of BC patients get worsened. Reduced LVEF was the predominant manifestation of both CTR‐CVT and late‐onset CTR‐CVT, which was then followed by an arrhythmia. The combination of LV GLS and c‐LVEF is better in the prediction of CTR‐CVT. Only the LV GLS at 3 months can predict the late‐onset CTR‐CVT.

## AUTHOR CONTRIBUTIONS

**Zhiyue Liu:** Data curation (equal); writing – original draft (equal). **Mei Liu:** Data curation (equal); project administration (equal). **Xiaorong Zhong:** Project administration (equal). **Yupei Qin:** Data curation (equal). **Ting Liang:** Data curation (equal). **Ting Luo:** Project administration (equal). **Xi Yan:** Project administration (equal). **Zhuoqin Tang:** Software (equal). **Xi Wang:** Resources (equal). **Shichu Liang:** Resources (equal); software (equal). **Qian Li:** Data curation (equal); software (equal); visualization (equal). **Xiaomiao Ruan:** Formal analysis (equal); investigation (equal). **Wenfeng He:** Data curation (equal). **He Huang:** Supervision (lead); Writing – review & editing (equal).

## FUNDING INFORMATION

HH is funded by Key Research and Development Programs of the Science and Technology Department of Sichuan Province. (Grant no. 2019YFS0414).

## CONFLICT OF INTEREST STATEMENT

The authors declare that the research was conducted in the absence of any commercial or financial relationships that could be construed as a potential conflict of interest.

## ETHICS APPROVAL STATEMENT

This study was approved by the Biomedical Ethics Committee of West China Hospital, Sichuan University (Approval Number: 20180517), and all patients provided informed consent.

## Data Availability

The data that support the findings of this study are available from the corresponding author, He Huang, upon reasonable request.
